# Modest contribution of metabolomic data to genomic prediction of breeding values for feed conversion ratio in pigs

**DOI:** 10.1186/s12711-026-01064-7

**Published:** 2026-06-24

**Authors:** Xiangyu Guo, Mark Henryon, Vinzent Börner, Tage Ostersen, Ole F. Christensen

**Affiliations:** 1 Breeding and Genetics, Sector for Pig, Danish Agriculture & Food Council, Copenhagen V, 1609 Denmark; 2 GHPC consulting and services PTY LTD, Armidale, 2350 Australia

## Abstract

**Background:**

Feed efficiency is an economically important but costly trait to measure in pig breeding. Previous studies have shown that integrating metabolomic data, such as proton nuclear magnetic resonance (¹H NMR) - derived metabolomic profiles, into genomic prediction models can improve the accuracy of estimated breeding values (EBVs) - for example, using a univariate metabolomic-genomic best linear unbiased prediction (MGBLUP) model for malting quality traits in barley and for average daily gain (ADG) in pigs using NMR-based metabolomic features (MFs). In this study, we extend this approach to predict feed conversion ratio (FCR) in pigs. We tested two hypotheses: (1) incorporating NMR metabolomic data into a univariate MGBLUP model increases the accuracy of EBVs for FCR compared with a univariate genomic BLUP (GBLUP) model, and (2) a bivariate MGBLUP model that jointly analyses FCR and the correlated trait ADG further improves EBV accuracy compared with a univariate MGBLUP model. We tested these hypotheses using an offspring-validation design, allowing prediction of EBVs for animals lacking individual FCR records.

**Methods:**

The experimental population comprised 8,174 Duroc pigs (4,027 males from a test station and 4,147 females from breeding herds). To evaluate the accuracy of EBVs for FCR, males with FCR records were used as the training population, and females without FCR records served as the validation population. These validation females had offspring with recorded FCR, and EBV accuracy was assessed by correlating their EBVs with the corrected phenotypes of their offspring.

**Results:**

Incorporating metabolomic data into the univariate MGBLUP model generated EBVs for FCR that were 2.3% more accurate than EBVs from the univariate GBLUP model (0.394 vs. 0.385). The bivariate MGBLUP model that jointly analysed FCR and ADG generated EBVs for FCR that were 1.2% more accurate than EBVs from the bivariate GBLUP model (0.432 vs. 0.427).

**Conclusions:**

The increases in accuracy were modest and statistically insignificant, reflecting limitations in the current metabolomic data, such as single-time-point sampling. Even so, the observed improvements suggest that metabolomic information may provide complementary information beyond genomic data. With further refinements in sampling strategies and genetic models, metabolomics could contribute to improving prediction accuracy in animal breeding.

**Supplementary Information:**

The online version contains supplementary material available at 10.1186/s12711-026-01064-7.

## Background

Feed efficiency, defined as the ability of an animal to convert feed into body weight, is a major determinant of production costs in commercial pig farming, because feed typically accounts for between 60 and 70% of the overall costs [1, 2]. Improving feed efficiency is of primary focus in genetic pig breeding programs due to its substantial implications for both economic profitability and environmental sustainability [3, 4]. Though feed efficiency is critical for pig production, it is a challenge to improve it genetically, since it is expensive to measure, requiring specialized equipment such as individual feeders. For example, in the DanBred breeding system, only boars sent to a centralized test station are measured for feed efficiency. Pigs in the breeding herds are not measured. Furthermore, feed efficiency is a result of a complex interaction among genetic factors, dietary composition, and management practices, and is influenced by a network of biological pathways involving metabolism, digestion, and growth [5, 6]. A better modelling of these complex interactions may be beneficial for genetic improvement of feed efficiency.

Metabolomics, the comprehensive analysis of small-molecule metabolites in biological samples, provides a high-dimensional dataset that reflects the current physiological state of an animal [7]. In livestock, metabolomic data are commonly generated using two major analytical platforms: proton nuclear magnetic resonance (^¹^H NMR) spectroscopy and mass-spectrometry (MS) - based methods. NMR typically produces spectral regions or bins, often referred to as metabolomic features (MFs), whereas MS generally quantifies chemically identified metabolites. These approaches differ in data structure and resolution, which is important for their downstream use in genetic evaluation. Compared with MS-based approaches, ¹H NMR spectroscopy offers superior analytical reproducibility, minimal sample preparation, and inherently quantitative measurements, making it particularly suitable for large-scale studies and genetic analyses that require high comparability across samples and batches [8]. Across livestock species metabolomic profiling has been applied to several sample types. For example, one study explored metabolomic profiles in both serum and urine from livestock, highlighting the potential of ^¹^H NMR spectroscopy to offer unique insights for monitoring health and physiological conditions critical to production performance [9]. Among various tissues, blood-based metabolomics provides a comprehensive and readily accessible snapshot of an animal’s metabolic status, as metabolites circulating in the blood integrate signals from multiple organs and reflect the overall physiological condition of the animal [9].

Several studies have already explored the use of metabolomic data in pigs. Rohart et al. [10] used ^¹^H NMR - based metabolomic profiles from growing pigs to predict production traits, including feed intake, growth, and carcass characteristics, and demonstrated that NMR - derived MFs can explain up to 30% of the phenotypic variation in these traits [10]. Other pig studies have used metabolomics to identify biomarkers for fertility and breed differences. For example, metabolite profiling of pig seminal plasma has been used to identify potential biomarkers for sperm resilience and ejaculate quality [11], and plasma and serum metabolomic profiles have been used to distinguish between Italian Large White and Italian Duroc pigs and relate metabolite patterns to genetic background [12]. Recent studies have specifically investigated the genetic architecture of metabolomic profiles in pigs. Bovo et al. [13] studied metabolomics and genomics in more than 1300 pigs from Large White and Duroc breeds, estimating heritabilities for 188 plasma metabolites and identifying 97 metabolite quantitative trait loci (mQTL) associated with 126 metabolites, thereby constructing a comprehensive catalog of genetic factors influencing the pig blood metabolome. Similarly, Ponsuksili et al. [14] studied the fetal plasma metabolome in pigs, reporting genomic heritability estimates for over 1000 metabolites and identifying 448 significant mQTL, with lipids showing particularly strong genetic regulation. Earlier work by the same group also revealed numerous QTL for blood clinical-biochemical traits in pigs, highlighting genes involved in cholesterol metabolism and other pathways [15]. These findings collectively demonstrate that MFs are under genetic control and can capture genetic variation relevant to production traits, providing a strong rationale for integrating metabolomic data into genetic evaluation models. These results together show that NMR - based metabolomic data can capture biologically meaningful information related to economically important traits in pigs.

Different approaches have been investigated to utilize metabolomic profiles for animal breeding. One approach focuses on identifying biomarkers that are associated with economically important traits, such as fertility, growth, or product quality, thereby linking specific metabolites to phenotypic and genetic variation [11, 12, 16]. Another approach is to integrate metabolomic information into a genetic model together with phenotypic and genomic data. For example, our previous study on malting quality traits in barley adapted the multi-omics model and methods by Christensen et al. [4] in the realm of plant breeding [17]. The multi-omics model, known as the metabolomic-genomic best linear unbiased prediction (MGBLUP) model, increased the accuracy of estimated breeding values (EBVs) for malting quality traits in barley compared with the standard genomic best linear unbiased prediction (GBLUP) model, with absolute increases in prediction accuracy of up to 0.03 [17]. Motivated by these results, we extended the use of MGBLUP to pigs by integrating blood metabolomic profiles into genomic predictions for average daily gain (ADG). In pigs, however, the improvement in prediction accuracy was limited, with EBV accuracy increasing by no more than about 0.02 (from 0.60 to 0.62) [18]. In that study, ADG was analyzed using a univariate model, whereas multi-trait models generally have the potential to improve accuracy by exploiting genetic correlations among traits, which is an advantage that is particularly relevant for feed efficiency traits, such as feed conversion ratio (FCR), which are not recorded on all animals [19].

Therefore, MGBLUP holds promise for incorporating NMR-based metabolomic data into genetic evaluation for feed efficiency in pigs. In the present study, we assessed the potential of integrating ^¹^H NMR - derived blood metabolomic data into genetic evaluation for FCR, which is the standard measure of feed efficiency in the DanBred pig breeding system. In doing so, we tested the hypotheses that (1) incorporating metabolomic data into a univariate MGBLUP model increases the accuracy of EBV for FCR compared with a univariate GBLUP model, and (2) a bivariate MGBLUP model that jointly analyses FCR and the correlated trait ADG further increases EBV accuracy compared with a univariate MGBLUP model. We tested these hypotheses through an offspring validation scheme, which allows us to validate female pigs in DanBred breeding herds without own FCR records, but having offspring raised in the test station with FCR records.

## Methods

The MGBLUP model was fitted to FCR in DanBred Duroc pigs to evaluate the accuracy of EBVs in an offspring validation scheme. Both the univariate model of FCR and the bivariate model including FCR and ADG were fitted.

### Experimental design

The experimental population consisted of 8,174 Duroc pigs, including 4,027 males and 4,147 females, which were recorded for FCR and ADG as part of the DanBred genetic evaluation. All pigs were recorded for ADG, but only the males, which were reared at a central test station were recorded for FCR. Females, which were reared in five different breeding herds, were not recorded for FCR. To evaluate the accuracy of EBVs for FCR, males with recorded FCR data were designated as the training data, while a subgroup consisting of 500 females with offspring recorded for FCR served as the validation population. There were no records of FCR on these females in breeding herds, but approximately 50% of the males at the test station were weaned in these breeding herds. The genetic connectedness between the training and validation populations was ensured through the extensive use of artificial insemination, resulting in shared sires across breeding herds and between the breeding herds and the test station, thereby providing a robust genetic link for offspring-based validation of EBVs.

### Data

The data used in this study consisted of phenotypes of FCR (FCR = feed intake/weight gain) and ADG (ADG = weight gain/days) in the body weight interval of 30–100 kg, pedigree information, genomic information, and records of NMR MFs.

Four populations were defined in this study: (1) Training Population including 4,027 males from the test station, (2) Validation Population including 500 females from breeding herds, (3) Offspring Population including 969 males recorded for FCR and offspring of the females in the Validation Population, and (4) Whole Population including all pigs (108,130) from the training population, validation population, and offspring population, as well as other pigs born from 2018 to 2024. A summary of phenotypic data for each population is shown in Table 1.

**Table 1:** Summary of phenotypic data for each population

Population	Nid	Trait	Nobs	Min	Mean	Max	Variance	S.D.
WP	108,130	ADG	108,130	783.50	1260.72	1951.50	15209.97	123.33
WP	108,130	FCR	13,336	1.45	1.98	2.51	0.02	0.13
TP	4,027	ADG	4,027	1011.00	1284.14	1524.00	6886.01	82.98
TP	4,027	FCR	4,025	1.54	1.95	2.38	0.01	0.11
VP	500	ADG	500	898	1242.98	1564.60	13788.32	117.42
VP	500	FCR	0	NA	NA	NA	NA	NA
OP	969	FCR	969	1.49	1.95	2.37	0.01	0.12

*WP* Whole population, *TP* Training population, *VP* Validation population, *OP* Offspring population, *ADG* Average daily gain, *FCR* Feed conversion ratio, *Nid* Number of individuals, *Nobs* Number of non-missing observations

As part of the DanBred breeding program, all pigs in the breeding system are routinely genotyped for single-nucleotide polymorphism (SNP) markers using the 50 K GGP-Porcine Illumina Bead SNP. After applying quality control measures to the SNP genotypes, a total of 33,960 SNPs remained. The quality control process ensured that each SNP had a unique and known position on one of the 18 autosomes or the X chromosome, based on Sus Scrofa build 11.1, had a minimum allele frequency greater than 0.001, showed no significant deviation from Hardy-Weinberg equilibrium (p-value < 0.0000001; only tested for SNPs on autosomes), and had a proportion of detected Mendelian errors below 0.001. Additionally, for each pig, a minimum genotype call rate of 80% was required, and individuals with parentage errors were re-genotyped. Imputation was carried out using FImpute 3 [20]. In the present study, we used only genotypes for the 8,174 pigs in the experimental population for which NMR metabolomic profiles were available.

Blood samples for NMR analysis were collected once from each of the 8,174 pigs at the end of their 30–100 kg performance-test period, when animals reached approximately 100 kg. The samples were sent to the Swedish NMR Centre at the University of Gothenburg, Sweden. The NMR analyses were conducted in four batches over a period from mid-2021 to early 2023.

### Preparation for NMR analysis

The NMR data is the same as the data used in our previous study, and therefore, the NMR analysis and data processing will only be briefly described. Further details are available in [18].

#### NMR analysis

Plasma samples were thawed for 45 min, mixed with an equal volume of buffer, and transferred to SampleJet tubes. Sample preparation and cooling were automated using a SamplePro Tube L robot. ^1^H NMR data acquisition followed Bruker IVDr standard protocols [21], including quality assurance checks and one-dimensional experiments using 600 MHz Bruker Avance III HD spectrometer with a cooled SampleJet sample changer.

#### Metabolomic features and NMR data processing

Our analysis focused on 32,697 NMR intensities, spanning a range from 0 parts per million (ppm) to 10 ppm. Data processing, implemented via a custom MATLAB script by Haggart et al. in 2019 [22], included exponential apodization function, Fourier transformation, alignment to the glucose anomeric proton doublet at ~ 5.23 ppm, automatic phasing, and baseline correction. Water peaks (4.4 ppm to 4.9 ppm) and a standard region (0 ppm to 0.04 ppm) were excluded, yielding 28,119 MFs. Probabilistic quotient normalization (PQN) [23] and final spectral alignment via the icoshift algorithm [24, 25] ensured robust data integrity of analyses.

PQN was applied to correct for sample-specific dilution differences, and the icoshift alignment was used to adjust small chemical-shift variations across spectra. These steps reduce technical noise and improve comparability between samples. All MFs were retained for analysis.

### Statistical models and methods

Both bivariate and univariate models were applied in this study. The bivariate models fitted FCR and ADG, while the univariate models fitted FCR. Prediction accuracies generated by the MGBLUP models were compared with the GBLUP models.

#### Bivariate models

##### GBLUP

The bivariate GBLUP for FCR fitted together with ADG was:

1 $$\begin{aligned}\:\left[\begin{array}{c}{\mathbf{y}}_{\mathrm{F}}\\\:{\mathbf{y}}_{\mathrm{A}}\end{array}\right]&=\left[\begin{array}{cc}{\mathbf{X}}_{\mathrm{F}}&\:0\\\:0&\:{\mathbf{X}}_{\mathrm{A}}\end{array}\right]\left[\begin{array}{c}{\mathbf{b}}_{\mathrm{F}}\\\:{\mathbf{b}}_{\mathrm{A}}\end{array}\right]+\left[\begin{array}{cc}{\mathbf{Z}}_{{\mathbf{g}}_{\mathrm{F}}}&\:0\\\:0&\:{\mathbf{Z}}_{{\mathbf{g}}_{\mathrm{A}}}\end{array}\right]\left[\begin{array}{c}{\mathbf{g}}_{\mathrm{F}}\\\:{\mathbf{g}}_{\mathrm{A}}\end{array}\right]\\&+\left[\begin{array}{c}0\\\:{\mathbf{Z}}_{{\mathbf{p}}_{\mathrm{A}}}{\mathbf{p}}_{\mathrm{A}}\end{array}\right]+\left[\begin{array}{c}0\\\:{{\mathbf{Z}}_{{\mathbf{l}}_{\mathbf{A}}}\mathbf{l}}_{\mathrm{A}}\end{array}\right]+\left[\begin{array}{c}{\mathbf{e}}_{\mathrm{F}}\\\:{\mathbf{e}}_{\mathrm{A}}\end{array}\right]\end{aligned}$$

where the subscript *F* represents FCR and the subscript *A* represents ADG; **y** is a vector of observations of a trait, $\:\mathbf{b}$ denotes a vector of fixed effects, including herd-section-year-month, birth parity, and regressions on initial weight and litter size at birth, $\:\mathbf{g}$ is a vector of additive genetic effects explained by genomic marker genotypes, $\:\mathbf{l}$ is a vector of litter effects, $\:\mathbf{p}$ is a vector of pen effects, and $\:\mathbf{e}$ is a vector of residual effects, capturing variation that remain unexplained by the other model effects. Matrices $\:\mathbf{X},\:{\mathbf{Z}}_{\mathrm{g}},\:{\mathbf{Z}}_{\mathrm{p}}$, and $\:{\mathbf{Z}}_{\mathrm{l}}$ are corresponding incidence matrices for $\:\mathbf{b}$, $\:\mathbf{g}$, $\:\mathbf{p}$ and $\:\mathbf{l}$, respectively. The random effects were assumed to follow independent multivariate normal distributions:

$$\:\left[\begin{array}{c}{\mathbf{g}}_{\mathrm{F}}\\\:{\mathbf{g}}_{\mathrm{A}}\end{array}\right]\sim\mathrm{M}\mathrm{V}\mathrm{N}\left(0,\left[\begin{array}{cc}{{\upsigma\:}}_{{\mathrm{g}}_{\mathrm{F}}}^{2}&\:{{\upsigma\:}}_{{\mathrm{g}}_{\mathrm{F}},{\mathrm{g}}_{\mathrm{A}}}\\\:{{\upsigma\:}}_{{\mathrm{g}}_{\mathrm{F}},{\mathrm{g}}_{\mathrm{A}}}&\:{{\upsigma\:}}_{{\mathrm{g}}_{\mathrm{A}}}^{2}\end{array}\right]\otimes\mathbf{G}\right)$$

$$\:\left[\begin{array}{c}{\mathbf{p}}_{\mathrm{A}}\\\:{\mathbf{l}}_{\mathrm{A}}\\\:{\mathbf{e}}_{\mathrm{F}}\\\:{\mathbf{e}}_{\mathrm{A}}\end{array}\right]\sim\mathrm{M}\mathrm{V}\mathrm{N}\left(0,\:\left[\begin{array}{c}{{\upsigma\:}}_{{\mathrm{p}}_{\mathrm{A}}}^{2}\\\:0\\\:0\\\:0\end{array}\begin{array}{c}0\\\:{{\upsigma\:}}_{{\mathrm{l}}_{\mathrm{A}}}^{2}\\\:0\\\:0\end{array}\begin{array}{c}0\\\:0\\\:{{\upsigma\:}}_{{\mathrm{e}}_{\mathrm{F}}}^{2}\\\:{{\upsigma\:}}_{{\mathrm{e}}_{\mathrm{F}},{\mathrm{e}}_{\mathrm{A}}}\end{array}\begin{array}{c}0\\\:0\\\:{{\upsigma\:}}_{{\mathrm{e}}_{\mathrm{F}},{\mathrm{e}}_{\mathrm{A}}}\\\:{{\upsigma\:}}_{{\mathrm{e}}_{\mathrm{A}}}^{2}\end{array}\right]\otimes\mathbf{I}\right)$$

Here, matrix $\:\mathbf{G}$ is an additive genomic relationship matrix, computed using the VanRaden method 1 [26], $\:\mathbf{I}$ is an identity matrix, $\:{{\upsigma\:}}_{\mathrm{g}}^{2}$, $\:{{\upsigma\:}}_{\mathrm{p}}^{2}$, $\:{{\upsigma\:}}_{\mathrm{l}}^{2}$, $\:{{\upsigma\:}}_{\mathrm{e}}^{2}$ are the variance parameters of genetic, pen, litter and residual effects, respectively, and $\:{{\upsigma\:}}_{{\mathrm{g}}_{\mathrm{F}},{\mathrm{g}}_{\mathrm{A}}}$ and $\:{{\upsigma\:}}_{{\mathrm{e}}_{\mathrm{F}},{\mathrm{e}}_{\mathrm{A}}}$ are the covariance parameters between FCR and ADG for, respectively, genetic effects and residual effects.

Pen and litter effects were included only for ADG, not for FCR. Models with a random pen and litter effects for FCR were attempted, but the variances explained by these effects were negligible. Therefore these two effects were omitted from the final model.

##### MGBLUP

Second, a bivariate MGBLUP was fitted. The method aligns with Christensen et al. [27], where it was shown that EBVs in the metabolomic-genomic model can be obtained by solving two mixed model equation systems successively, where each of these systems correspond to a linear mixed model.

The first step is a bivariate model for FCR and ADG:

2 $$\begin{aligned}\:\left[\begin{array}{c}{\mathbf{y}}_{\mathrm{F}}\\\:{\mathbf{y}}_{\mathrm{A}}\end{array}\right]&=\left[\begin{array}{cc}{\mathbf{X}}_{\mathrm{F}}&\:0\\\:0&\:{\mathbf{X}}_{\mathrm{A}}\end{array}\right]\left[\begin{array}{c}{\mathbf{b}}_{1\mathrm{F}}\\\:{\mathbf{b}}_{1\mathrm{A}}\end{array}\right]+\left[\begin{array}{cc}{\mathbf{Z}}_{{\mathbf{g}}_{\mathrm{F}}}&\:0\\\:0&\:{\mathbf{Z}}_{{\mathbf{g}}_{\mathrm{A}}}\end{array}\right]\left[\begin{array}{c}{\mathbf{g}}_{1\mathrm{F}}\\\:{\mathbf{g}}_{1\mathrm{A}}\end{array}\right]\\&+\left[\begin{array}{cc}{\mathbf{Z}}_{{\mathbf{m}}_{1\mathrm{F}}}&\:0\\\:0&\:{\mathbf{Z}}_{{\mathbf{m}}_{1\mathrm{A}}}\end{array}\right]\left[\begin{array}{c}{\mathbf{m}}_{1\mathrm{F}}\\\:{\mathbf{m}}_{1\mathrm{A}}\end{array}\right]\\&+\left[\begin{array}{c}0\\\:{{\mathbf{Z}}_{{\mathbf{p}}_{\mathrm{A}}}\mathbf{p}}_{1\mathrm{A}}\end{array}\right]+\left[\begin{array}{c}0\\\:{{\mathbf{Z}}_{{\mathbf{l}}_{\mathbf{A}}}\mathbf{l}}_{1\mathrm{A}}\end{array}\right]+\left[\begin{array}{c}{\mathbf{e}}_{1\mathrm{F}}\\\:{\mathbf{e}}_{1\mathrm{A}}\end{array}\right]\end{aligned}$$

where **y**, **b**, **g**, **p**, **l**, **e**, **X**, **Z** are defined as for the Eq. (1) with subscript 1 referring to the first step of the Eq. (2), and $\:\mathbf{m}$ is the vector of metabolomic effects on phenotype, $$\:\left[\begin{array}{c}{\mathbf{m}}_{\mathrm{F}}\\\:{\mathbf{m}}_{\mathrm{A}}\end{array}\right]\sim\mathrm{M}\mathrm{V}\mathrm{N}\left(0,\left[\begin{array}{cc}{{\upsigma\:}}_{{\mathrm{m}}_{\mathrm{F}}}^{2}&\:{{\upsigma\:}}_{{\mathrm{m}}_{\mathrm{F}},{\mathrm{m}}_{\mathrm{A}}}\\\:{{\upsigma\:}}_{{\mathrm{m}}_{\mathrm{F}},{\mathrm{m}}_{\mathrm{A}}}&\:{{\upsigma\:}}_{{\mathrm{m}}_{\mathrm{A}}}^{2}\end{array}\right]\otimes\mathbf{Q}\right)$$, with $\:\mathbf{Q}$ being the metabolomic similarity matrix defined as described in Guo et al. [8], $\:{{\upsigma\:}}_{\mathrm{m}}^{2}$ is the variance parameter of metabolomic effects, and $\:{{\upsigma\:}}_{{\mathrm{m}}_{\mathrm{F}},{\mathrm{m}}_{\mathrm{A}}}$ is the covariance parameter between the metabolomic effects for FCR and ADG. Let matrix $\:\mathbf{M}$ be a *p* × *q* matrix of NMR intensities with *p* = 8,174 (equal to number of samples) and *q* = 28,119 (equal to number of MFs). To minimize variations arising from experimental sources and signal intensities, $\:\mathbf{Q}=\frac{\mathbf{M}{\mathbf{M}}^{\mathbf{{\prime\:}}}}{\mathrm{q}}$, where columns of $\:\mathbf{M}$ were scaled to unit variance [28].

The second step is a univariate model for FCR only:

3 $$\:{\widehat{\mathbf{m}}}_{\mathbf{F}}={\mathbf{X}}_{2}{\mathbf{b}}_{2}+{\mathbf{Z}}_{{\mathrm{g}}_{2}}{\mathbf{g}}_{2}+{\mathbf{e}}_{2}$$

where $\:{\widehat{\mathbf{m}}}_{\mathbf{F}}$ is the vector of predicted metabolomics effects from the first step for FCR, and other vectors and matrices are defined similarly to those in the Eq. (1). The distribution of random effects are $\:{\mathbf{g}}_{2}\sim \; N\left(0,\mathbf{G}{\sigma\:}_{{g}_{2}}^{2}\right)$ and $\:{\mathbf{e}}_{2}\sim \; N\left(0,\mathbf{I}{\sigma\:}_{{e}_{2}}^{2}\right)$, with the different random effects assumed independent of each other, according to Christensen et al. [27], and based on the assumption of MFs having the same variances.

#### Univariate models

To compare with bivariate models, univariate GBLUP and MGBLUP models were also applied for FCR.

4 $$\:{\mathbf{y}}_{\mathrm{F}}={\mathbf{X}}_{\mathrm{F}}{\mathbf{b}}_{\mathrm{F}}+{\mathbf{Z}}_{{\mathbf{g}}_{\mathrm{F}}}{\mathbf{g}}_{\mathrm{F}}+\:{\mathbf{e}}_{\mathrm{F}}$$

5 $$\begin{aligned}\:{\mathbf{y}}_{\mathrm{F}}&={\mathbf{X}}_{\mathrm{F}}{\mathbf{b}}_{1\mathrm{F}}+{\mathbf{Z}}_{{\mathbf{g}}_{\mathrm{F}}}{\mathbf{g}}_{1\mathrm{F}}\\&+{\mathbf{Z}}_{{\mathbf{m}}_{1\mathrm{F}}}{\mathbf{m}}_{1\mathrm{F}}+\:{\mathbf{e}}_{1\mathrm{F}}\end{aligned}$$

6 $$\:{\widehat{\mathbf{m}}}_{1\mathbf{F}}={\mathbf{X}}_{2}{\mathbf{b}}_{2}+{\mathbf{Z}}_{{\mathrm{g}}_{2}}{\mathbf{g}}_{2}+{\mathbf{e}}_{2}$$

where all the effects in the univariate models are defined as for the bivariate models.

### Estimation of (co)variance components

The full data set was used to estimate variance components in GBLUP and MGBLUP by restricted maximum likelihood using the APEX software (www.ghpc.ai).

For GBLUP, the genomic variance is $\overline{\left(\overline{G}_d-\overline{G} \right)}\sigma_g^2$ and $\sigma_P^2 =\overline{\left(\overline{G}_d-\overline{G} \right)}\sigma_g^2+\sigma_e^2$, so that the genomic heritability of GBLUP is $h^2_{\mathrm{GBLUP}}=\overline{\left(\overline{G}_d-\overline{G} \right)}\sigma_g^2/\sigma_P^2$, where $\overline{G_d}$ is the average of diagonal elements in the $\:\mathbf{G}$ matrix and $\overline{G}$ is the average of all elements of the $\:\mathbf{G}$ matrix, which is equal to 0.

For MGBLUP, $\sigma_{P_1}^2 =\overline{\left(\overline{Q}_d-\overline{Q} \right)}\sigma_m^2+\overline{\left(\overline{G}_d-\overline{G} \right)}\sigma_{g_1}^2 + \sigma_{e_1}^2$, where $\overline{Q_d}$ is the average diagonal of the $\:\mathbf{Q}$ matrix, and $\overline{Q}$ is the average of the $\:\mathbf{Q}$ matrix, which is equal to 0. Here, the direct genomic variance is $\overline{\left(\overline{G}_d-\overline{G} \right)}\sigma_{g_1}^2$, the direct heritability is $h^2_d=\overline{\left(\overline{G}_d-\overline{G} \right)}\sigma_{g_1}^2/\sigma_{P_1}^2$, and the metabolomic variance ratio is $c_m^2=\overline{\left(\overline{Q}_d-\overline{Q} \right)}\sigma_m^2/\sigma_{P_1}^2$. Furthermore, according to Christensen et al. [27], heritability of the metabolomic intensities can be obtained from second step of MGBLUP, i.e. here $h^2_m=\overline{\left(\overline{G}_d-\overline{G} \right)}\sigma_{g_2}^2/\left[\overline{\left(\overline{G_d}-\overline{G} \right)}\sigma_{g_2}^2 + \sigma_{e_2}^2 \right]$, and therefore according to Christensen et al. [27], the genomic heritability based on the MGBLUP model is: $\:{\mathrm{h}}_{\mathrm{M}\mathrm{G}\mathrm{B}\mathrm{L}\mathrm{U}\mathrm{P}}^{2}={\mathrm{c}}_{\mathrm{m}}^{2}{\mathrm{h}}_{\mathrm{m}}^{2}+{\mathrm{h}}_{\mathrm{d}}^{2}$.

Genetic, metabolomic and residual correlations were calculated from corresponding covariances and variances.

### Estimated breeding values

In the GBLUP model, EBVs correspond to the predicted genetic effects, $\:\widehat{\mathbf{g}}$. In contrast the MGBLUP model yields EBVs in two components: $\:{\widehat{\mathbf{g}}}_{1}$, the genomic effects estimated jointly with metabolomic effects in the first step of MGBLUP, and $\:{\widehat{\mathbf{g}}}_{2}$, the remaining genomic effects estimated in the second step of MGBLUP. The EBVs were calculated as the sum of EBVs from first and second steps of MGBLUP, following the derivation in Christensen et al. [27], which is $\:\widehat{\mathbf{a}}={\widehat{\mathbf{g}}}_{1}+{\widehat{\mathbf{g}}}_{2}$.

### Predictive abilities and accuracy

To quantify the predictive ability of EBVs for individuals in the validation population, we computed the correlation between the vector of phenotypes from the offspring population corrected for all fixed effects/regressions and the vector of EBVs generated by GBLUP and MGBLUP. The calculation of breeding values for females was based on a training population including 4,027 males with their own records of ADG and FCR, genotypes and MFs, and a validation population including 500 females with their own records of ADG (but not FCR), genotypes and MFs. The calculation of corrected phenotypes of offsprings was based on a population including 108,130 pigs with ADG records and 13,336 of them having FCR records, and a pedigree including 123,513 pigs.

Converting predictive ability for offspring into accuracy of EBVs for their dams, the following formula was used: $\:\mathrm{a}\mathrm{c}\mathrm{c}\mathrm{u}\mathrm{r}\mathrm{a}\mathrm{c}\mathrm{y}=\sqrt{\frac{{\left(\mathrm{p}\mathrm{r}\mathrm{e}\mathrm{d}\mathrm{i}\mathrm{c}\mathrm{t}\mathrm{i}\mathrm{v}\mathrm{e}\:\mathrm{a}\mathrm{b}\mathrm{i}\mathrm{l}\mathrm{i}\mathrm{t}\mathrm{y}\right)}^{2}}{\mathrm{s}\mathrm{c}\mathrm{a}\mathrm{l}\mathrm{i}\mathrm{n}\mathrm{g}\:\mathrm{f}\mathrm{a}\mathrm{c}\mathrm{t}\mathrm{o}\mathrm{r}}}$, where the: $\:\mathrm{s}\mathrm{c}\mathrm{a}\mathrm{l}\mathrm{i}\mathrm{n}\mathrm{g}\:\mathrm{f}\mathrm{a}\mathrm{c}\mathrm{t}\mathrm{o}\mathrm{r}=\:\frac{0.5\times\:{{\upsigma\:}}_{\mathrm{a}}^{2}}{0.75\times\:{{\upsigma\:}}_{\mathrm{a}}^{2}+{{\upsigma\:}}_{\mathrm{e}}^{2}}$ with $\:{{\upsigma\:}}_{\mathrm{a}}^{2}$ and $\:{{\upsigma\:}}_{\mathrm{e}}^{2}$ being the additive genetic and residual variance from the pedigree-based model, used for the calculation of corrected phenotypes.

Differences in predictive ability and accuracy of EBVs between the four models of interest were assessed using a Hotelling-Williams t-test [29, 30] at level 5%.

## Results

In this study, variance components were first estimated. Then the breeding values were predicted using GBLUP and MGBLUP bivariate and univariate models. Accuracies of EBVs were evaluated by offspring validation.

### Estimated variance components

**Fig. 1:**
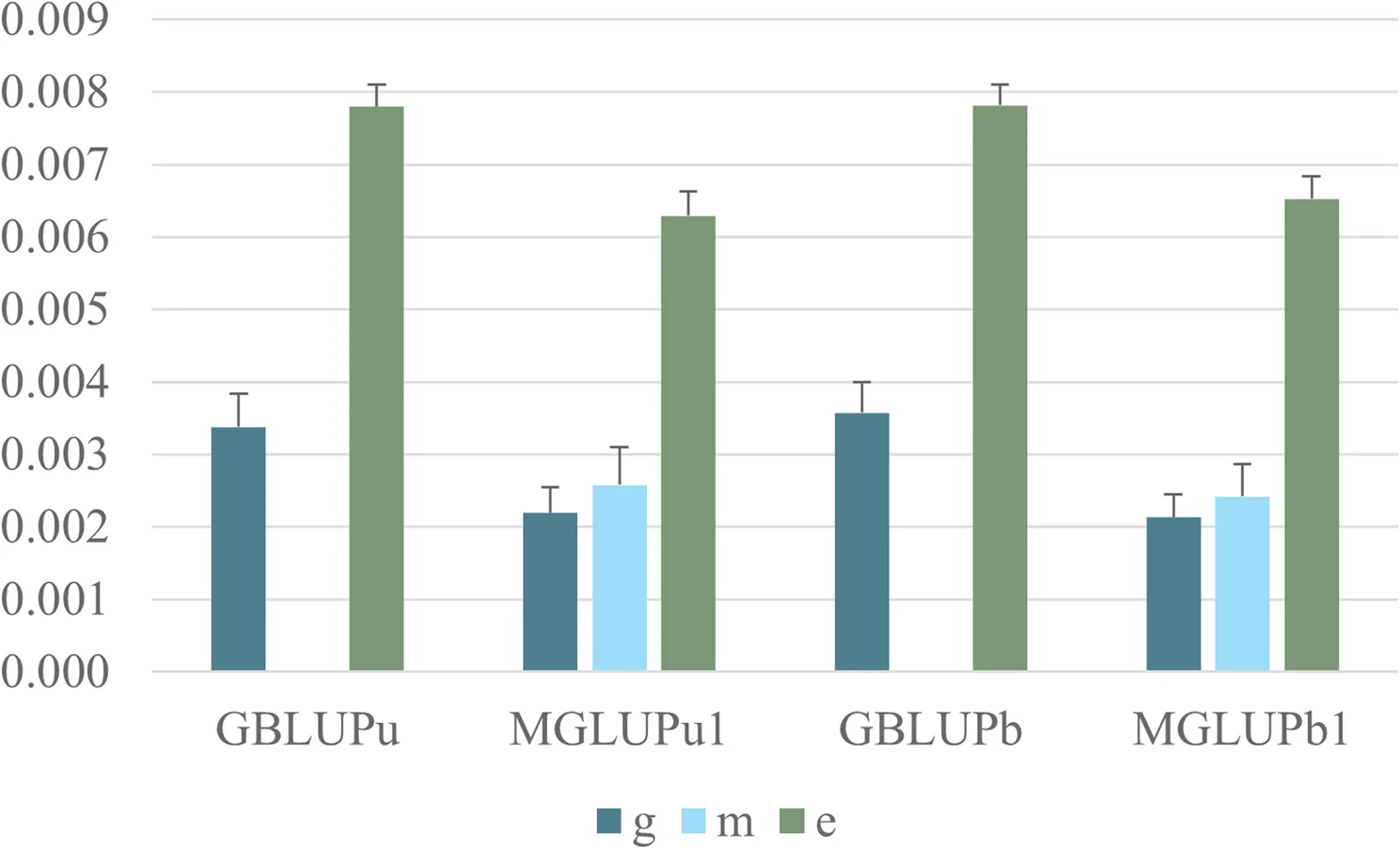
Estimated variance components together with standard errors from different models. X-axis: model and variance components; y-axis: estimates of variances. g: genomic variance; m: metabolomic variance; e: residual variance. GBLUPu: univariate model with genomic relationship matrix; MGBLUPu1: first step of univariate model with genomic relationship matrix and metabolomic similarity matrix; GBLUPb: bivariate model with genomic relationship matrix; MGBLUPb1: first step of bivariate model with genomic relationship matrix and metabolomic similarity matrix

Figure 1 shows the estimated variance components for FCR in the GBLUP and MGBLUP models (step 1) fitted as univariate and bivariate models (parameter estimates for ADG are shown in Additional file 1 Figure S1). Including metabolomic data into the model decreased the direct genomic variance compared to the genomic variance in GBLUP. For the univariate model, the decrease was 35.1%, while for the bivariate model, the decrease was 40.3%. For the GBLUP models, the genomic variance increased by 5.7% when using the bivariate model compared to the univariate model. For the MGBLUP models, genomic variance decreased by 2.8% when using the bivariate model compared to the univariate model.

The decreases in residual variances when including metabolomic data into the models were similar to those for direct genomic variances, i.e. there is a decrease of 19.3% when using the MGBLUP univariate model compared to the GBLUP univariate model, and a decrease of 16.5% when using the MGBLUP bivariate model compared to the GBLUP bivariate model.

The metabolomic variances ($\:{{\upsigma\:}}_{\mathrm{m}}^{2}$) decreased by 6.3% when using the MGBLUP bivariate model compared to the MGBLUP univariate model, which is similar to the decreases in genomic variances.

For total phenotypic variances, integrating metabolomic data into models yielded decreases of 0.1% for the univariate model and 2.7% for the bivariate model. Introducing ADG into models increased the total phenotypic variances of FCR by 1.9% for the GBLUP model and 0.1% for MGBLUP model.

These parameter estimates resulted in estimates of heritability of 0.30 for the GBLUP univariate model and 0.31 for the GBLUP bivariate model. Further, it resulted in a direct heritability of 0.20 for the MGBLUP univariate model, and 0.19 for the MGBLUP bivariate model. The proportion of variance explained by the metabolome was 0.22 for the MGBLUP univariate model, and 0.23 for the MGBLUP bivariate model. For MGBLUP, the heritability of metabolomic intensities ($\:{h}_{m}^{2}$) was 0.31. The total heritability of FCR in the MBLUP model ($\:{\mathrm{h}}^{2}={\mathrm{c}}_{\mathrm{m}}^{2}{\mathrm{h}}_{\mathrm{m}}^{2}+{\mathrm{h}}_{\mathrm{d}}^{2}$) was 0.27 for the univariate model and 0.26 for the bivariate model.

In the bivariate models, the genetic correlation was − 0.08 ± 0.045 in GBLUP and − 0.47 ± 0.04 in MGBLUP, the residual correlation was − 0.69 ± 0.01 in GBLUP and − 0.80 ± 0.01 in MGBLUP, and the metabolomic correlation was − 0.15 ± 0.06 in MGBLUP.

### Predictive ability and accuracy of EBVs

Table 2 shows the predictive ability and accuracy of EBVs using the offspring validation scheme for GBLUP and MGBLUP. For the univariate models, the predictive ability was 0.181 for univariate GBLUP and 0.185 for univariate MGBLUP, corresponding to 2% increase when adding metabolomic data. For the bivariate model, the predictive ability was 0.201 for bivariate GBLUP and 0.203 bivariate MGBLUP, corresponding to 1% increase when adding metabolomic data. When converted to accuracy of EBVs, incorporating metabolomic data into the univariate MGBLUP model generated EBV for FCR that were 2.3% more accurate than those from the univariate GBLUP model (0.394 vs. 0.385). Similarly, the bivariate MGBLUP model that jointly analysed FCR and ADG produced EBVs that were 1.2% more accurate than those from the bivariate GBLUP model (0.432 vs. 0.427). When comparing the bivariate and univariate MGBUP models, the accuracy of EBVs increased by 9.6% (0.432 vs. 0.394). The bivariate MGBLUP also achieved the highest overall accuracy (0.432) among all models tested, although none of these improvements were statistically significant.

**Table 2:** Predictive ability and accuracy of estimated breeding values of feed conversion ratio from different models

Model	Predictive ability	Accuracy
GBLUPu	0.181	0.385
MGBLUPu	0.185	0.394
GBLUPb	0.201	0.427
MGBLUPb	0.203	0.432

*GBLUPu* Univariate model with genomic relationship matrix, *MGBLUPu* Univariate model with genomic relationship matrix and metabolomic similarity matrix, *GBLUPb* Bivariate model with genomic relationship matrix, *MGBLUPb* Bivariate model with genomic relationship matrix and metabolomic similarity matrix

## Discussion

Our findings support our hypotheses that (i) MGBLUP generates more accurate EBVs for FCR compared to GBLUP, and (ii) the EBVs from bivariate MGBLUP are more accurate than univariate MGBLUP. MGBLUP improved accuracy of EBV for FCR by about 2% and 1% with univariate and bivariate models, suggesting that metabolites provide additional information beyond pedigree and genomics. However, the 10% increase in accuracy for FCR with bivariate MGBLUP compared to univariate MGBLUP probably resulted from the genetic covariance between FCR and ADG, rather than from metabolomic information. This was evidenced by a similar increase in accuracy when moving from univariate to bivariate GBLUP, where metabolites were not included. Although the increases in accuracy were modest and statistically insignificant, we remain encouraged by metabolomics for two reasons. First, they represent marginal improvements in models already incorporating pedigree and genomic data. Second, they were achieved using an immature methodology, highlighting opportunities to further refine the approach. These refinements could include identifying the relevant tissue types, and sampling times that influence FCR, as well as improving genetic models that include metabolomic information and establishing optimal methods for extracting concentrations of relevant metabolites. While current methods are not yet ready for practical application in breeding schemes, our findings do represent an important step toward developing an advanced selection criterion. Although metabolomic information shows promise, its practical application also depends on economic feasibility. Currently, the per-sample cost of NMR-based metabolomic profiling is comparable to that of routine SNP-chip genotyping, but it requires blood sampling which limits its cost-effectiveness given the modest gains in prediction accuracy observed in this study. Future reductions in metabolomics costs or the development of cheaper targeted metabolite panels may improve the economic viability of incorporating metabolomic data into breeding programs. So, we believe metabolomics has significant potential to improve prediction accuracy in breeding programs for both plants and animals, with its role expected to grow as we deepen our understanding of the relationship between metabolites and trait expression.

Comparing estimates of variances for FCR in MGBLUP and GBLUP, the direct genetic and residual variances in MGBLUP were smaller than those in GBLUP, regardless of whether univariate or bivariate models were used. This suggests that the variance attributed to metabolomic effects in GBLUP included both genetic and environmental components. Based on the parameter estimates from MGBLUP, the direct heritability was approximately 0.2, which was slightly smaller than the proportion of variance explained by metabolomics. Combined with the heritability of MFs also being around 0.2, this led to an overall heritability of 0.26. These results indicate that while the MFs are heritable and capture part of the variation in the trait, most of the variation they capture is environmental, and only a small proportion of the genetic variance is mediated through the metabolome. Similar results were observed in our previous study on ADG, where the proportion of genetic variance mediated by metabolome was even smaller than that in the current study for FCR [18]. Moreover, the type of variation captured by metabolomic data may vary across different study contexts. In our pig studies, metabolomic data appeared to predominantly reflect environmental variation, which may help reduce residual noise and allow pedigree and genomic effects to better capture genetic signals. To complement the variance components analysis, we also estimated the correlation parameters between FCR and ADG using bivariate models. We noted large changes in the correlations between FCR and ADG when introducing metabolomic data. In particular, the genetic correlation in GBLUP is around zero, whereas the residual genetic correlation in MGBLUP is around − 0.5. The changes in genetic and residual correlations from GBLUP to MGBLUP are likely due to the inclusion of metabolomic effects, which incorporate both genetic and environmental components. These results further confirm that metabolomic data capture a mixture of genetic and environmental variation. In practical terms, this means that metabolomic data may help account for environmental noise in prediction models, enabling genetic effects to be estimated more precisely. Despite the substantial difference in genetic correlation estimates, the correlation between EBVs for FCR obtained from GBLUP and MGBLUP was 0.98, indicating that animal rankings for selection remain largely consistent.

Multivariate MGBLUP has not been investigated in previous studies. In our study, the bivariate MGBLUP model improved accuracy of EBV by about 10% compared with univariate MGBLUP, consistent with the results described earlier. Importantly, the improvement from the bivariate framework exceeded that obtained by adding metabolomic data alone, underscoring the benefit of leveraging information from genetically correlated traits. This may be because part of the information in their own metabolomic data was already captured by jointly modelling FCR and ADG in the bivariate framework, limiting the additional contribution of metabolomics. Therefore, although bivariate MGBLUP was successfully applied in this study, its improvement in prediction accuracy over bivariate GBLUP remained limited.

Our modest improvements in prediction accuracy for FCR are consistent with our previous pig study on ADG, where combining genomic and metabolomic data also produced limited gains [21]. In that study, the inclusion of metabolomic data increased the prediction accuracy of ADG only marginally, being equivalent to approximately 6–10% of the gain achieved when the animal’s own phenotype was added. These findings closely mirror the small improvements observed here for FCR when metabolomic information was incorporated. The consistency across traits suggests that, under the current sampling strategy and use of biological material, metabolomic profiles provide only modest additional information beyond genomic data. The similar level of improvement for both ADG and FCR suggests that metabolomic data currently provide only a small but consistent contribution to improving prediction accuracy for growth-related and feed-efficiency traits in pigs. While ADG and FCR are strongly correlated, extending these findings to other feed efficiency traits that are phenotypically independent of growth, such as residual feed intake (RFI), remains less straightforward. Unlike FCR, RFI is defined to be phenotypically independent of growth traits, and its genetic and biological regulation may therefore differ from that of ADG or FCR. Consequently, the extent to which metabolomic profiles that are informative for ADG or FCR would also improve prediction accuracy for RFI is uncertain. It is possible that MFs associated with RFI reflect different physiological processes, which may limit the direct transferability of the present results. Therefore, although our findings highlight the potential of metabolomics for traits genetically correlated with ADG, generalizing these conclusions to alternative feed efficiency criteria such as RFI will require dedicated studies explicitly targeting such traits.

This pattern likely reflects the characteristics of both the trait and the biological samples used, as blood metabolomic profiles collected at the end of the test period may not contain metabolites that reliably predict production traits such as FCR or ADG. In contrast, a previous study on malting quality traits in barley showed around a 10% gain in prediction accuracy when metabolomic data from malt samples were included [17]. This comparison highlights two important factors that influence utility of metabolomic data: the biological relevance of the sampled tissue and the timing of collection relative to the expression of the target trait. For pigs, sampling tissues directly involved in nutrient metabolism, such as liver, muscle, or gut, at biologically meaningful time points may provide stronger associations with feed efficiency traits. Moreover, the type of variation captured by metabolomic data can differ across species and contexts. In pigs, metabolomics appeared to primarily reflect environmental variation, helping to reduce residual noise; while in barley, metabolomic profiles captured a larger proportion of genetic variation, directly contributing to improved prediction accuracy. Together, these findings suggest that metabolomic data can enhance genomic prediction either by accounting for environmental effects or by serving as proxies for genetic influences, depending on how closely the metabolome reflects the genetic architecture of the target trait.

The limited improvement in prediction accuracy of FCR when animals had their own metabolomic data likely reflects both the nature of the trait and the timing of sampling. FCR and ADG are cumulative traits measured over extended production period (approximately 30–100 kg body weight), while blood samples were taken only once, at the end of the testing. This temporal mismatch likely limited the capacity of the metabolomic data to represent the dynamic biological processes influencing the trait throughout the entire growth period. Based on these insights, future studies should consider incorporating metabolomic data from biologically relevant tissues collected at multiple time points and refine MGBLUP models to more clearly separate genetic and environmental variance components. The potential impact of sampling time on predictive performance has been highlighted in other omics contexts. For example, Maltecca et al. showed that fecal microbiome composition at different time points (weaning, mid-test, and later test stages) formed distinct microbial populations and provided varying contributions to prediction accuracy depending on when the microbiome was sampled relative to trait measurement [31]. This suggests that omics sampled at multiple biologically meaningful time points may better capture dynamic biological processes and improve prediction of cumulative traits, compared with a single end-of-test sample. Such refinements are expected to enhance the predictive power and practical utility of multi-omics approaches in animal breeding.

Beyond sampling time, several methodological aspects of the MGBLUP framework warrant further discussion, particularly regarding how potential redundancies among MFs are handled. The decision to retain all 28,119 MFs, despite known correlations among them, follows the conceptual framework of the MGBLUP model: construction of the metabolomic similarity matrix $\:\mathbf{Q}=\frac{\mathbf{M}{\mathbf{M}}^{\mathbf{{\prime\:}}}}{\mathrm{q}}$ is analogous to the genomic relationship matrix $\:\mathbf{G}$, which utilizes all SNPs regardless of linkage disequilibrium [26]. In both cases, the multivariate structure is exploited through the covariance matrix rather than feature selection, and the mixed model framework accounts for correlations among predictors [27]; high correlations among features are reflected in $\:\mathbf{Q}$ and are explicitly modeled during variance component estimation. Similar approaches retaining complete spectral data have been used in other multi-omics studies [17, 28], and whether these fully and optimally addresses redundancy is an active area of methodological research. Furthermore, evidence from other multi-omics studies suggests that feature selection approaches may capture less total variance than full-spectrum methods, as biologically relevant signals can be distributed across correlated features. For example, Riedelsheimer et al. [32] demonstrated that using all 130 metabolites (without selection) achieved high prediction accuracies (0.60–0.80) for complex traits in maize, supporting the use of complete metabolic profiles. Future studies could explore whether dimension reduction techniques, such as principal component analysis applied prior to $\:\mathbf{Q}$ matrix construction, offer advantages in model interpretability, while ensuring that such approaches do not discard features capturing important environmental variation [18].

## Conclusions

The increases in accuracy of EBV were modest and statistically insignificant. Variance component estimates revealed that MFs capture predominantly environmental variation, with the temporal mismatch between single time-point sampling of metabolomic data and cumulative trait measurement of FCR likely limiting predictive utility. These findings suggest that while metabolomics holds theoretical promise, its practical utility for feed efficiency traits in pigs remains limited with current sampling strategies. The study identifies specific limitations, particularly sampling timing and tissue selection, that can guide future research. Whether refined protocols or alternative platforms could yield greater benefits remains an open question, but at present, the additional value of NMR-based metabolomic data for predicting FCR breeding values appears modest.

## Supplementary Information

Supplementary Material 1.

## Data Availability

Data supporting this study are not publicly available due to data protection policy by private company. Please contact corresponding author for possibility.
