# Supplementary material for: Modest contribution of metabolomic data to genomic prediction of breeding values for feed conversion ratio in pigs
Source: Genet Sel Evol. 2026 Jun 24;58:44. doi: 10.1186/s12711-026-01064-7 (PMC13491720; doi:10.1186/s12711-026-01064-7)
Supplement: Supplementary file 1 [file 12711_2026_1064_MOESM1_ESM.docx]

**Modest contribution of metabolomic data to genomic prediction of breeding values for feed conversion ratio in pigs**

Xiangyu Guo^1*^, Mark Henryon^1^, Vinzent Börner^2^, Tage Ostersen^1^, Ole F. Christensen^1^

^1^ Breeding and Genetics, Sector for Pig, Danish Agriculture & Food Council, 1609 Copenhagen V, Denmark

^2^ GHPC consulting and services PTY LTD, Armidale, 2350, Australia

^*^Corresponding author

E-mail addresses:

XG: xguo@lf.dk

MH: [mahe@lf.dk](mailto:mahe@lf.dk)

VB: vinzentboerner@ghpc.ai

TO: tao@lf.dk

OFC: olch@lf.dk

**Additional file 1**

**Figure S1.** Estimated variance components of average daily gain together with standard errors from different models.

x-axis: model and variance components; y-axis: estimates of variances

g: genomic variance; m: metabolomic variance; p: pen variance; l: litter variance; e: residual variance

GBLUPu: univariate model with genomic relationship matrix; MGBLUPu1: first step of univariate model with genomic relationship matrix and metabolomic similarity matrix; GBLUPb: bivariate model with genomic relationship matrix; MGBLUPb1: first step of bivariate model with genomic relationship matrix and metabolomic similarity matrix.
